# Adulteration of Essential Oils: A Multitask Issue for Quality Control. Three Case Studies: *Lavandula angustifolia* Mill., *Citrus limon* (L.) Osbeck and *Melaleuca alternifolia* (Maiden & Betche) Cheel

**DOI:** 10.3390/molecules26185610

**Published:** 2021-09-16

**Authors:** Francesca Capetti, Arianna Marengo, Cecilia Cagliero, Erica Liberto, Carlo Bicchi, Patrizia Rubiolo, Barbara Sgorbini

**Affiliations:** Dipartimento di Scienza e Tecnologia del Farmaco, Università degli Studi di Torino, Via Pietro Giuria 9, I-10125 Turin, Italy; francesca.capetti@unito.it (F.C.); arianna.marengo@unito.it (A.M.); cecilia.cagliero@unito.it (C.C.); erica.liberto@unito.it (E.L.); carlo.bicchi@unito.it (C.B.); patrizia.rubiolo@unito.it (P.R.)

**Keywords:** adulteration of essential oils, *Lavandula angustifolia*, *Citrus limon* (ex. Citrus × bergamia), *Melaleuca alternifolia*, chiral analysis

## Abstract

The quality control of essential oils (EO) principally aims at revealing the presence of adulterations and at quantifying compounds that are limited by law by evaluating EO chemical compositions, usually in terms of the normalised relative abundance of selected markers, for comparison to reference values reported in pharmacopoeias and/or international norms. Common adulterations of EO consist of the addition of cheaper EO or synthetic materials. This adulteration can be detected by calculating the percent normalised areas of selected markers or the enantiomeric composition of chiral components. The dilution of the EO with vegetable oils is another type of adulteration. This adulteration is quite devious, as it modifies neither the qualitative composition of the resulting EO nor the marker’s normalised percentage abundance, which is no longer diagnostic, and an absolute quantitative analysis is required. This study aims at verifying the application of the two above approaches (i.e., normalised relative abundance and absolute quantitation) to detect EO adulterations, with examples involving selected commercial EO (lavender, bergamot and tea tree) adulterated with synthetic components, EO of different origin and lower economical values and heavy vegetable oils. The results show that absolute quantitation is necessary to highlight adulteration with heavy vegetable oils, providing that a reference quantitative profile is available.

## 1. Introduction

Essential oils (EO) are complex mixtures of volatile compounds that are characterised by important biological activities for the plant itself and for humans who have learned to exploit their properties over the centuries. The ISO norm 9235:2013 defines an EO as a “…product obtained from a natural raw material of plant origin, by steam distillation, by mechanical processes from the epicarp of citrus fruits, or by dry distillation, after separation of the aqueous phase—if any—by physical processes” [1]. The European Pharmacopoeia terms an EO as “an Odorous product, usually of complex composition, obtained from a botanically defined plant raw material by steam distillation, dry distillation, or a suitable mechanical process without heating. Essential oils are usually separated from the aqueous phase by a physical process that does not significantly affect their composition” [2]. In both definitions, it is clear that only products obtained by steam/hydrodistillation can be named EO, while products obtained by different extraction procedures involving the use of solvents must be defined as extracts. EO are mainly characterised by the presence of terpenes/terpenoids and phenolic compounds (i.e., phenylpropanoids), that derive from the mevalonate/methyl erithrytol and shikimic acid pathways, respectively.

The chemical composition of an EO is usually expressed in the literature in terms of the relative percentage abundance (i.e., % area) or normalised percentage abundance (i.e., norm % areas) [3]. Only a few papers have reported the true quantitation of EO, as determining this is considered difficult and time-consuming.

The quality control of EO is necessary to guarantee their safe use, as well as to detect adulterations and fraud. Unfortunately, the adulteration of EO is not uncommon along supply chains, thus generating concerns in the EO industry. EO can often be adulterated via the addition of cheaper EO (e.g., sweet orange added to bitter orange, corn mint added to peppermint or lavandin added to lavender) or via the addition of cheap synthetic materials (e.g., synthetic linalool and linalyl acetate added to bergamot EO) [4]. This type of adulteration can be detected quite easily via the determination of the normalised percentage areas of selected markers. Moreover, since biosynthesis in plants is stereochemically guided and terpenes/terpenoids are generally chiral compounds with a specific enantiomeric composition [5,6], chiral marker compounds become diagnostics for detecting the adulteration of essential oils via the addition of synthetic volatile compounds. Enantiomeric recognition is therefore also necessary to improve the quality control and uncover fraud and adulteration via the addition of cheap synthetic materials or volatiles from other sources to EO [4].

Dilution with vegetable oils, resulting in a reduction in scent, is another type of EO adulteration. Vegetable oils are selected, as they are relatively cheap and because they present a density and texture that are similar to those of EO [4]. This type of adulteration is quite devious, as it modifies neither the qualitative composition of the EO nor the relative percentage abundance of the marker compounds. However, the dilution of the final product interferes with the EO sensory and biological properties, in addition to committing commercial fraud. In this case, the normalised percentage area is no longer diagnostic, and a true quantitative analysis is required, provided that acceptable reference quantitative data are available.

To the best of the authors’ knowledge, several papers describing strategies for revealing EO adulterations that occur via the addition of cheaper EO or synthetic compounds have been reported in the literature and have recently been reviewed [4,7,8]. Conversely, there are few papers that describe approaches to reveal the addition of vegetable oils to dilute EO [9,10,11]. The most applied technique is infrared (IR) spectroscopy coupled with a multivariate analysis. However, the precise identification of vegetable oil is often difficult due to the signal overlap of similar molecules [11]. Very recently, Truzzi et al. introduced a new method based on ^13^C-NMR spectroscopy to recognise adulterant vegetable oils in EO; the method does not require additional data elaboration with a multivariate analysis [12].

This study evaluates and compares different approaches to detect adulterations of three representative EO (i.e., bergamot, lavender and tea tree EO)—in particular, the determination of the normalised percentage areas and/or the enantiomeric composition of selected markers and the true absolute quantitative analysis. These three EO were selected due to their global market impact, which includes a constant increase both in terms of production and worldwide market share [13]. Both conventional and chiral GC analyses were performed, and the latter was combined with HS-SPME sampling to avoid damage to the chiral column degradation due to non-eluted residues of vegetable oil.

## 2. Results and Discussion

In the routine quality control of EO, tens of samples are analysed every year, and usually, some borderline samples may be found. These EO demand special attention.

EO adulteration can successfully be testified only when a suitable reference profiling of genuineness obtained with the appropriate analytical methods (i.e., % areas through conventional GC analysis, enantiomeric composition through enantioselective GC analysis and true quantitation) is available either from international organisations (e.g., pharmacopoeias or ISO norms) or built in-house by analysing a consistent number of certified genuine samples.

This study is fully in line with this strategy and consists of: (1) the creation of the reference profiles of the EO under investigation, including their enantiomeric recognition, via the analysis of different batches of genuine EO (20 samples of each EO), (2) the analysis of EO adulterated via the addition of synthetic volatile compounds or cheap EO and (3) the analysis of adulterated EO via the addition of vegetable oil requiring a true quantitation to detect the dilution.

This study focuses on some commercially available EO samples that were found to be at the limit of acceptance when compared to the pharmacopoeia monograph—namely, two lavender, one bergamot and two tea tree (TTO) EO.

### 2.1. Reference Chemical Profiles of Genuine Essential Oils

**Figure 1:**
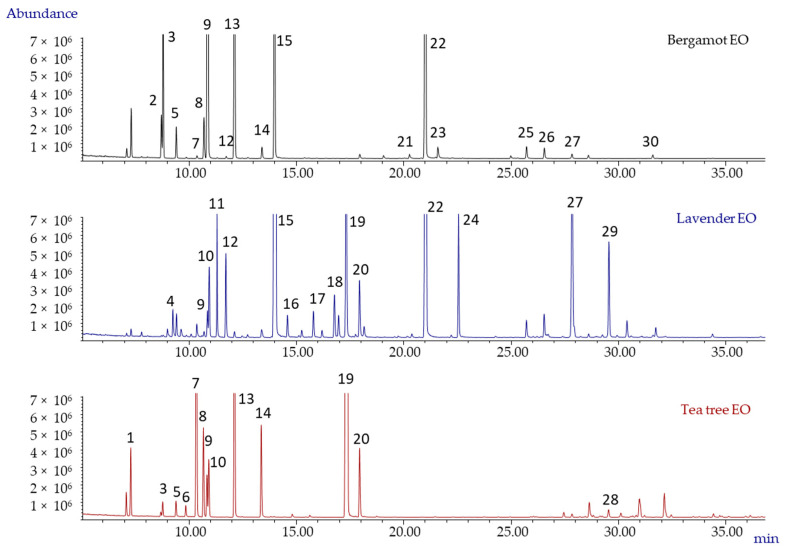
GC-MS patterns of genuine bergamot, lavender and TTO EO. Legend: (1) α-pinene, (2) sabinene, (3) β-pinene, (4) 3-octanone, (5) β-myrcene, (6) α-phellandrene, (7) α-terpinene, (8) *p*-cymene, (9) limonene, (10) 1,8-cineole, (11) *cis*-β-ocimene, (12) *trans*-β-ocimene, (13) γ-terpinene, (14) α-terpinolene, (15) linalool, (16) 1-octen-3-yl acetate, (17) camphor, (18) lavandulol, (19) 4-terpineol, (20) α-terpineol, (21) neral, (22) linalyl acetate, (23) geranial, (24) lavandulyl acetate, (25) neryl acetate, (26) geranyl acetate, (27) *trans*-β-caryophyllene, (28) aromadendrene, (29) *trans*-β-farnesene and (30) β-bisabolene. For the analysis conditions, see the main text.

Generally, the reference profile should include the minimum, average and maximum percent normalised area values for each selected marker compound, which should be calculated using a sufficient (significantly representative) number of controlled genuine samples. Figure 1 shows the GC-MS patterns of genuine bergamot, lavender and tea tree EO. These EO were characterised mostly by monoterpenes/monoterpenoids and sesquiterpenes/sesquiterpenoids; each EO profile presented some predominant compounds (e.g., linalool and linalyl acetate in bergamot and lavender EO and 4-terpineol and γ-terpinene in the tea tree EO), together with other relatively minor compounds.

Table 1 reports the composition of the investigated EO in terms of the normalised relative abundance (minimum, maximum and average % areas, together with the standard deviation values) of the characteristic marker compounds determined by analysing twenty genuine batches for each EO. Table 1 also reports the Italian/European Pharmacopoeia % area ranges.

All of the analysed batches were in agreement with the Italian or European Pharmacopoeia, as it is evident from the minimum and maximum normalised % areas, respectively, and the ranges that were narrower than those of the pharmacopoeias for the selected markers, which indicates the high homogeneity of the selected samples. The average reference composition of bergamot EO was also in agreement with Verzera et al. [14], who analysed 1082 genuine samples.

Table 2 reports the percentage enantiomeric composition (EC%) of some representative chiral markers in the investigated genuine EO. They were determined using the same number of samples as those used to build up the reference profiles (*n* = 20). Tables S1–S3 in the Supplementary Materials report the EC% of all the enantiomeric compounds in the investigated genuine EO. The EC% values were calculated using the following formula:

EC% = [Area _enantiomer R or S_/Area _enantiomer R_ + Area _enantiomer S_] × 100

The results obtained for bergamot were compared to those reported by Mondello et al. [15], which were obtained via the analysis of about 100 genuine EO samples. The samples here used to build up the reference profile were all found to be genuine, as the EC% was perfectly superimposable with the literature data. Bergamot EO are characterised by a clear predominance of (*R*)-(−)-linalool and (*R*)-(−)-linalyl acetate versus (*S*)-enantiomers by 98.6% and 98.7%, respectively.

Furthermore, lavender EO are also characterised by a clear predominance of (*R*)-(−)-linalool and (*R*)-(−)-linalyl acetate versus S-enantiomers by 93.8% and 99.4%, respectively, which is confirmed by the literature data [18] and pharmacopoeia [1].

Different is the situation of TTO, where the three markers (i.e., limonene, α-terpineol and 4-terpineol) from Australia and China have similar normalised % abundances when analysed with conventional GC, therefore making it almost impossible to distinguish between them. The Australian TTO, however, presents a diagnostic enantiomeric ratio, the abundance of (*R*)-enantiomers of limonene and α-terpineol being remarkably higher than the (*S*)-form (i.e., 61.0% and 39.0% for (*R*)- and (*S*)-limonene and 75.8% and 24.2% for (*R*)- and (*S*)-α-terpineol), while 4-terpineol is mainly present in the (*S*)-form. Conversely, their EC% in Chinese tea tree EO are significantly different. These data indicate that enantiomeric recognition is therefore a diagnostic to distinguish the different origins, which, incidentally, also significantly characterise their economic value. The informative value of chiral recognition for TTO has also been recognised by ISO that included the enantiomeric ratio of 4-terpineol in the 2017 revision of ISO 4730 Standard [16] that specifies the enantiomeric ratio for 4-terpineol as (*S*)-(+) 67–71% and (*R*)-(−) 29–33%. In this case too, the results here reported for the investigated Australian samples are in agreement with both the ISO norm and the data of Wong et al. [17] for about 60 genuine samples.

### 2.2. Adulteration with Cheaper Essential Oils or Synthetic Compounds

To evaluate the genuineness of the investigated commercial EO, the genuine lavender and bergamot EO chosen to build up the reference profiling were first adulterated with synthetic linalool and linalyl acetate (spiked samples) [19] and the genuine samples of Australian TTO with Chinese TTO (mixed origins sample) (see Section 3 for details). Table 3 shows that the normalised relative abundances of linalool, linalyl acetate and α-terpineol increased in both the commercial and spiked samples and are borderline compared to the reference chemical profile, but this increase was not sufficient to decide a clear adulteration, since they still were within the reference range reported by the pharmacopoeias. Conversely, the TTO sample obtained by mixing the two origins (i.e., Australian and Chinese) did not show a significant variation in terms of the normalised relative abundance of 4-terpineol, probably because of the similar compositions of the two EO.

On the other hand, Table 2 shows that the enantiomeric composition dramatically changed—in particular, in bergamot samples, (*S*)-(+)-linalool increased from 0.4% to 24% and (*S*)-(+)-linalyl acetate from 0.3% to 10.9%. The same was true for lavender EO, where (*S*)-(+)-linalool was raised from 6.2% to 33.6% and (*S*)-(+)-linalyl acetate from 0.6% to 19.6% (See Table 2). In both cases, the EC% values exceeded the maximum reference values, clearly showing their adulterations.

**Figure 2:**
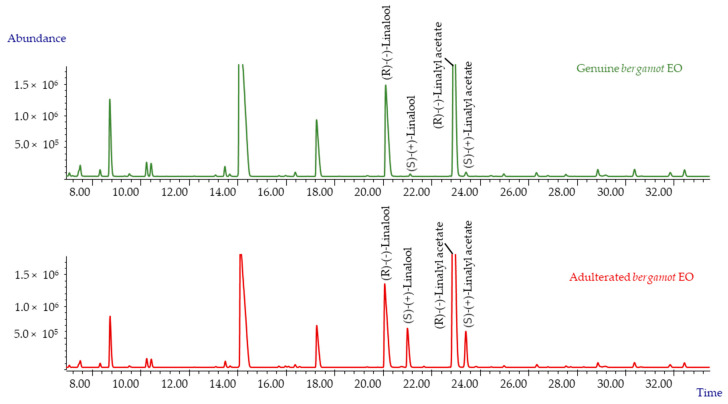
Linalool and linalyl acetate in a genuine and an adulterated bergamot EO analysed with a 2,3DE6TBDMS-β-CD chiral column phase.

This is also evident in Figure 2 reporting the chromatogram of linalool and linalyl acetate enantiomers in a genuine and in a spiked bergamot EO, both submitted to enantioselective GC with 2,3 di-O-ethyl-6-t-butyldimethyl silyl-β-cyclodextrin (2,3DE6TBDMS-β-CD) as the chiral selector.

A similar behaviour was observed for the mixture of Australian and Chinese TTO that resulted in a significant change in the enantiomeric composition of 4-terpineol, with a drop of EC% from the expected 68% to 69% indicated by ISO to 54.5% (see Table 2).

### 2.3. Commercial EO Adulterated with Vegetable Oils

**Figure 3:**
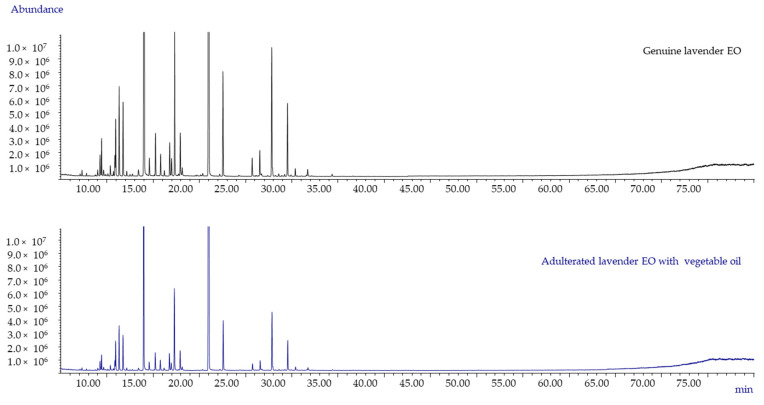
GC-MS patterns of both genuine and spiked on purpose lavender EO (analysed with an oven temperature program up to 300 °C).

As mentioned previously, the addition of vegetable oil produces a dilution of the EO that does not affect the qualitative GC profile but results in a decrease of the absolute amounts of the markers. Figure 3 shows the GC-MS patterns of both genuine and spiked on purpose lavender EO (analysed with an oven temperature program up to 300 °C). The GC patterns clearly indicate the absence of peaks due to vegetable oils, and it is perfectly superimposable from a qualitative point of view. However, the profile of the spiked sample presents peaks of lower intensity, maybe indicative of a dilution effect due to the presence of a heavy vegetable oil.

One of the commercial lavender EO samples (CL-2 EO) showed normalised peak area intensities of the selected markers significantly lower than those observed in genuine EO. The sample was submitted to the European Pharmacopoeia test to detect fatty oils [1] by putting a drop of the EO onto filter paper and a slight translucent spot after 24 h was evidenced.

The conventional GC and enantioselective GC analyses did not provide results suitable to measure the % of adulteration; therefore, a true quantitative analysis was required. A series of experiments were carried out to evaluate the reliability of this approach by adulterating the three oils of this study with different amounts of vegetable oils to confirm the % adulteration experimentally, although only one suspected commercial lavender sample (CL-2 OE) was the object of investigation. The data obtained for the commercial and adulterated EO were the same as those calculated in the genuine samples and already reported in Table 1 (data not shown, as it is redundant), driving us to perform a true quantitative analysis.

Table S4 reports the equations for the calibration curves that were obtained with an external standard approach and used to quantitate the marker compounds, together with their correlation coefficient and the selected range of concentrations. Table 4 reports the absolute concentrations of the selected markers in the genuine, the spiked and the commercial lavender oil EO under investigation.

These results showed that the commercial sample was adulterated about 40–50% with vegetable oil.

For a further confirmation, the absolute quantification was also carried out on the enantiomers of the markers of the CL-2 EO. The quantitative analysis was carried out with a Multiple Headspace Solid-Phase Microextraction (MHS-SPME) combined with an enantioselective GC-FID-MS to avoid the degradation of the cyclodextrin column performance due to non-eluted residues of heavy vegetable oil.

Table 5 reports the absolute concentrations of the enantiomers of linalool and linalyl acetate in the genuine, spiked with vegetable oil and CL-2 EO. The absolute concentrations of the single enantiomers clearly decreased with increasing the degree of adulteration, indicating the reliability of this approach to measure the EO adulteration with vegetable oils.

The results on the enantiomeric recognition of the CL-2 EO showed that it was adulterated between 40% and 50% with a heavy vegetable oil. The degree of adulteration of the sample analysed should be considered as indicative, since it was not possible to analyse the sample before the adulteration. This confirms the need for a representative genuine GC pattern.

## 3. Materials and Methods

### 3.1. Essential Oils, Standards and Materials

Genuine EO from botanically authenticated samples of *Citrus limon* (L.) Osbeck (ex *Citrus × bergamia* Risso et Poit, bergamot), *Lavandula angustifolia* Mill. (lavender) and *Melaleuca alternifolia* (Maiden & Betche) Cheel (Australian tea tree and Chinese tea tree) were supplied by Erboristeria Magentina (Poirino, Italy). The EO were obtained via steam distillation for lavender and tea tree EO and via cold expression for bergamot. Twenty different batches were considered for each EO. Some EO samples were also purchased in the local shops (commercial samples). Table 6 lists the EO used in this work, together with the specialised metabolites chosen as marker compounds and their target ions used for quantitation. The above genuine EO were spiked on purpose (spiked samples) to build a model of adulterations: (1) bergamot and lavender EO were supplemented with synthetic racemic linalool and linalyl acetate (9% and 11%, respectively, for bergamot EO and 27% of both linalool and linalyl acetate for lavender EO), (2) Australian TTO was mixed with 50% Chinese tea tree and (3) all the investigated EO were mixed with different amounts of sunflower vegetable oil (from 5% to 50%).

Pure standard commercially available samples (purity > 98%) of linalool, linalyl acetate, 4-terpineol, α-terpineol, (*R*)-(−)-linalool, (*S*)-(+)-linalool, (*R*)-(−)-linalyl acetate, (*S*)-(+)-linalyl acetate, (*R*)-(−)-4-terpineol, (*S*)-(+)-4-terpineol, (*R*)-(+)-α-terpineol and (*S*)-(−)-α-terpineol were purchased from Merck, Darmstadt, Germany. Tridecane (C13) was used as the internal standard and purchased from Merck. The alkane standard mixture (C9–C25) was prepared to calculate the retention indices (final concentration: 100 μg/mL). Cyclohexane was HPLC grade and supplied by Carlo Erba, Milano, Italy.

### 3.2. GC Analysis Conditions

GC-MS analyses were carried out on an Agilent 6890 GC unit coupled to an Agilent 5975 MSD (Agilent, Little Falls, DE, USA), equipped with a MPS-2 multipurpose sampler (Gerstel, Mülheim a/d Ruhr, Germany), using the following conditions: injector temperature: 230 °C; injection mode: split; ratio: 1/20; carrier gas: helium; flow rate: 1 mL min^−1^; columns: MEGA 5 (*d_f_* 0.25 µm, *d_c_* 0.25 mm and length 30 m) and 2,3-di-*O*-ethyl-6-*O*-t-butyldimethylsilyl-β-CD (2,3DE6TBDMS-β-CD) [20] (*d_f_* 0.25 µm, *d_c_* 0.25 mm and length 25 m) (Mega, Legnano, Milan, Italy). Temperature programs: for the MEGA 5 column from 50 °C (1 min) to 300 °C (5 min) at 3 °C min^−1^ and for the cyclodextrin column from 40 °C (1 min) to 220 °C (5 min) at 2 °C min^−1^. The marker compounds were identified by comparing their mass spectra and retention indices to those of authentic standards, to those that were commercial (Wiley, Nist and Adams) and/or homemade libraries or from the literature [21,22].

GC-FID analyses were carried out on a Shimadzu 2010 GC unit under the same conditions as reported above. The relative percentage compositions of the analysed EO were determined using GC-FID peak areas and applying response factors [3,23].

For the GC-MS and GC-FID analyses with the MEGA 5 column, the genuine, spiked and commercial essential oils were diluted in cyclohexane (5 mg/mL). Tridecane (C13) was used as the internal standard (final concentration in the dilution: 0.1 mg/mL). For the GC-MS and GC-FID analyses with the 2,3DE6TBDMS-β-CD column, the genuine, spiked and commercial EO were sampled using HS-SPME (for the conditions, see Section 3.4) to avoid cyclodextrin column degradation due to non-eluted residues of vegetable oil.

### 3.3. HS-SPME Sampling Conditions

For the genuine, spiked and commercial EO samples, 2 μL of the dilution in cyclohexane (5 mg/mL) were introduced in a 20-mL headspace vial, immediately hermetically closed with a PTFE-silicone septa and sampled for 20 min at room temperature (i.e., 25 °C) by HS-SPME. Stableflex carboxen/divinylbenzene/PDMS (CAR/DVB/PDMS) SPME fibres (2 cm long) from Supelco Co. (Bellafonte, PA, USA) were chosen.

After sampling, the fibre was automatically removed from the vapor phase (headspace) and introduced into the GC injector to allow the complete thermal desorption of the sampled analytes to occur in the GC column. Blank runs were carried out to verify the absence of carryover effects.

The fibre performance was periodically checked (every ten analyses) by adopting in-fibre external standardisation and by analysing a standard aqueous solution containing some of the selected markers (5 µL of a 2-mg mL^−1^ solution containing 4-terpineol, linalool and linalyl acetate) [24,25].

### 3.4. Quantitative Analysis

For the true quantitation of the selected markers in genuine, spiked and commercial EO, the external calibration method was chosen. Stock standard solutions were prepared via the addition of an aliquot of pure standard to an appropriate volume of cyclohexane (final concentration: 10 mg/mL). Suitable dilutions of each stock standard mixture were then prepared (final concentrations in the range of 5–0.1 mg/mL). The resulting stock and diluted solutions were supplemented with C13 (final concentration a dilution of 0.1 mg/mL), stored at 0 °C and renewed weekly. A calibration curve was built by analysing the above diluted solutions.

For the true quantitation of the selected enantiomers, the MHS-SPME approach was adopted by using the same diluted solutions that were sampled by MHS-SPME and using the total vaporization approach [26]. MHS-SPME is the most appropriate approach for volatile component quantitation in liquid or solid matrices that are sampled by the headspace. MHS-SPME is the SPME extension of the MHE-static HS that was developed by Kolb et al. [27,28], and is based on successive dynamic gas extraction from a single sample; the marker analyte peak area decays exponentially with the number of extractions, and the amount present initially in a given matrix (in this case, the EO) is represented by the sum of the areas from each extraction. The total area of the analyte(s) under investigation for quantitation is determined using the following equation:

(1) $$A_{T}=\sum_{i=1}^{\infty }A_{i}=\frac{A_{1}}{\left(1-e^{-q}\right)}=\frac{A_{1}}{\left(1-Q\right)}$$

where A_1_ is the analyte area after the first extraction, A_T_ is the total analyte area (derived from the sum of the areas from each extraction) and Q: e^−*q*^, −*q* is a constant calculated from the following linear regression analysis equation:

ln A_i_ = −*q* (i − 1) + ln A_1_ (2)

where A_i_ is the peak area obtained from the ith extraction. In everyday practice, a few extractions (generally, 3–5) are sufficient to obtain a reliable exponential equation that describes the analyte decay, from which the total area of the analyte in the sample can be successfully extrapolated. The analytes are then quantified using an external standard approach that is performed by submitting standard mixtures of selected markers at different concentrations to MHS-SPME. MHS-SPME can also be carried out under non-equilibrium conditions [29,30], provided that the sampling parameters are rigorously standardised and the amount of sample is suitable to give linear analyte decay(s).

Figure S1 in the Supplementary Materials shows the GC-MS-extracted ions of linalool (*m/z* = 71) in a bergamot EO, with three consecutive extractions from a sample (on the left) and its linear decay diagram (on the right).

## 4. Conclusions

The quality control of EO to highlight the presence of synthetic “naturally identical” substances or of less expensive EO can successfully be carried out by evaluating the chemical composition in terms of the normalised percentage area or true quantitation of the diagnostic markers to be compared with the reference data reported in pharmacopeia or ISO norm monographies. Most of the EO samples analysed in routine quality controls comply with the reference data (i.e., pharmacopoeia, ISO norm and in-house built reference profiling). Conversely, borderline EO samples require a more accurate evaluation to confirm or exclude their adulteration.

This study shows different approaches on how to deal with a successful quality control of borderline EO samples by using enantiomeric recognition and an absolute quantitative analysis of the selected marker compounds as a complement to the normalised relative abundances, thus making possible to highlight a number of EO adulterations with examples from real world samples. The approaches here adopted were based on gas chromatography, which is the technique of choice to characterise an EO. These methods afford to detect simultaneously both the presence of compounds deriving from different (cheaper) EO with a single GC run, to monitor the addition of synthetic racemic compounds by enantioselective and EO dilution with vegetable oil(s). Moreover, these approaches are very useful in routine quality control, because they do not require extra statistical elaboration, and analyses can be carried out using fast methods with the adoption of narrow bore GC columns, thanks to the repeatability of the separations ensured by the method translation software [31,32].

Adulteration with vegetable oils cannot be revealed using the above approach and requires an absolute quantitative analysis. This method of course requires reference concentration values obtained from the analysis of a consistent number of certified genuine EO samples. A quantitative analysis is often considered to be a complex time-consuming procedure; however, as clearly shown in this study, the quantitation of a limited number of selected markers is often sufficient to highlight an adulteration with vegetable oil(s); that is, the number of required analyses is rather low.

Direct methods based on spectroscopic methods have also been developed with success to deal with this problem: they include fluorescence spectroscopy [9], infrared spectroscopy [11] and Raman spectrometry [10]. These methods, however, generally require the use of multivariate statistical elaborations (principal component analysis and independent component analysis) or the building up of artificial neural networks, thus implying a further step of data processing. Very recently, Truzzi et al. reported a method based on NMR spectroscopy without a further statistical step not only able to detect the presence of an adulteration with vegetable oils but, also, to identify the added adulterant through its ^13^C-NMR fingerprint [12].

The availability of separation and direct methods (in particular, NMR) is an effective step ahead to monitor EO adulterations, since it is now possible to define their quality both in terms of the characteristic qualitative and quantitative marker composition and detection and identification of adulterants of low economic value.

In conclusion, the approaches adopted in this study, in combination with the methods based on NMR spectroscopy, open up a concrete possibility of identifying unambiguously EO adulterations by vegetable oil in quality control laboratories.

## Figures and Tables

**Table 1:** Normalised relative abundance (minimum, maximum and average normalised % area) of the marker compounds in bergamot, lavender and tea tree essential oils (number of samples: 20).

	***Citrus limon*** **(L.) Osbeck**							
	**Compounds**	**I^t^_s_exp**	**I^t^_s_lit**	**Min**	**Max**	**Average**	**σ**	**Ital. Ph.**
2	sabinene	976	976	0.8	1.0	0.9	0.1	0.5–2.0
3	β-pinene	978	980	5.6	6.9	6.5	0.4	5.0–10.0
5	β-myrcene	992	991	0.7	1.5	1.0	0.2	
7	α-terpinene	1018	1018	0.1	0.2	0.1	0.1	
8	*p*-cymene	1028	1026	0.2	0.7	0.4	0.2	
9	limonene	1031	1031	34.3	40.9	37.8	2.3	30.0–50.0
12	*trans*-β-ocimene	1051	1050	0.1	0.2	0.1	0.1	
13	γ-terpinene	1061	1062	5.3	8.0	6.7	0.9	6.0–18.5
14	α-terpinolene	1089	1088	0.3	0.6	0.3	0.1	
15	linalool	1100	1098	9.6	12.3	10.7	0.8	6.0–15.0
21	neral	1243	1240	0.1	0.3	0.2	0.1	
22	linalyl acetate	1264	1257	30.0	35.8	31.5	1.6	23.0–35.0
23	geranial	1273	1270	0.1	0.5	0.3	0.1	max 0.5
25	neryl acetate	1369	1365	0.3	0.8	0.5	0.1	
26	geranyl acetate	1386	1383	0.3	0.7	0.4	0.1	0.1–0.7
27	*trans*-β-caryophyllene	1419	1418	0.1	0.6	0.3	0.1	0.2–0.5
30	β-bisabolene	1510	1509	0.1	0.4	0.2	0.1	
	***Lavandula angustifolia*** **Mill.**							
	**Compounds**	**I^t^_s_exp**	**I^t^_s_lit**	**Min**	**Max**	**Average**	**σ**	**Eur. Ph.**
4	3-octanone	989	986	0.4	1.3	0.9	0.3	0.1–5.0
9	limonene	1031	1031	0.4	1.1	0.7	0.2	max 1.0
10	1,8-cineole	1033	1033	1.3	2.6	2.4	1.2	max 2.5
11	*cis*-β-ocimene	1041	1040	0.7	3.2	2.4	0.6	
12	*trans*-β-ocimene	1051	1050	0.2	2.2	1.8	0.5	
15	linalool	1100	1098	23.8	33.0	29.5	2.5	20.0–45.0
16	1-octen-3-yl acetate	1116	1110	0.4	1.1	0.7	0.2	
17	camphor	1147	1143	0.6	1.2	1.0	0.2	max 1.2
18	lavandulol	1171	1166	0.6	1.7	0.9	0.4	min 0.1
19	4-terpineol	1178	1177	2.6	6.0	3.6	1.0	0.1–8.0
20	α-terpineol	1191	1189	0.1	2.0	1.0	0.6	max 2.0
22	linalyl acetate	1264	1257	25.1	40.7	35.4	4.0	25.0–47.0
24	lavandulyl acetate	1293	1289	2.7	5.3	3.5	0.7	min 0.2
27	*trans*-β-caryophyllene	1419	1418	1.8	5.1	3.5	1.0	
29	*trans*-β-farnesene	1460	1458	1.2	5.2	2.5	1.0	
	***Melaleuca alternifolia*** **(Maiden & Betche) Cheel**							
	**Compounds**	**I^t^_s_exp**	**I^t^_s_lit**	**Min**	**Max**	**Average**	**σ**	**Eur. Ph.**
1	α-pinene	936	939	1.8	6.0	3.8	1.8	1.0–6.0
3	β-pinene	978	980	0.1	1.1	0.6	0.3	
5	β-myrcene	992	991	0.1	1.1	0.5	0.4	
6	α-phellandrene	1004	1005	0.0	0.6	0.3	0.2	
7	α-terpinene	1018	1018	7.7	10.5	9.2	0.7	5.0–13.0
8	*p*-cymene	1028	1026	0.7	3.6	2.1	0.8	0.5–12.0
9	limonene	1031	1031	0.8	3.7	2.5	1.0	0.5–4.0
10	1,8-cineole	1033	1033	1.9	7.1	3.9	1.5	max 15.0
13	γ-terpinene	1061	1062	14.3	22.4	19.2	2.4	10.0–28.0
14	α-terpinolene	1089	1088	2.0	4.7	3.1	0.8	1.5–5.0
19	4-terpineol	1178	1177	32.4	47.1	40.5	3.4	min 30.0
20	α-terpineol	1191	1189	2.6	7.1	5.0	1.4	1.5–8.0
28	aromadendrene	1439	1439	0.1	5.8	1.3	1.4	max 7.0

I^t^_s_exp: experimental programmed temperature retention index; I^t^_s_lit: tabulated programmed temperature retention index.

**Table 2:** Percent enantiomeric composition (EC%) of some representative markers in genuine and adulterated bergamot (commercial bergamot EO: CB-1 EO), lavender (commercial lavender EO: CL-1 EO) and TTO (commercial tee tree oil: CT-1 EO) EO compared to the literature data (*n* = 20).

			**Bergamot EO**			**Lavender EO**		
	**I^t^sexp**	**I^t^slit**	**Genuine**	**Adulterated** **CB-1 EO**	**Reference Values** [15]	**Genuine**	**Adulterated** **CL-1 EO**	**Reference Values** [1]
(*R*)-(−)-linalool	1181	1174	99.6	76.0	99.4–99.7	93.8	66.4	
(*S*)-(+)-linalool	1196	1189	0.4	24.0	0.3–0.6	6.2	33.6	max 12%
(*R*)-(−)-linalyl acetate	1233	1231	99.7	89.1	99.7–99.9	99.4	80.4	
(*S*)-(+)-linalyl acetate	1243	1237	0.3	10.9	0.1–0.3	0.6	19.6	max 1%
			**Chinese TTO**			**Australian TTO**		
	**I^t^sexp**	**I^t^slit**	**Genuine**			**Genuine**	**Adulterated** **CT-1 EO**	**Reference Values** [16,17]
(*R*)-(−)-4-terpineol	1258	1253	57.8			30.8	45.5	
(*S*)-(+)-4-terpineol	1250	1248	42.2			69.2	54.5	67.4–69.6
(*R*)-(+)-α-terpineol	1317	1309	77.0			75.8	76.7	71.0–78.0
(*S*)-(−)-α-terpineol	1302	1296	23.0			24.2	23.3	

**Table 3:** Normalised relative % abundance of linalool, linalyl acetate, 4-terpineol and α-terpineol in genuine, spiked and commercial samples of bergamot, lavender and TTO EO obtained with conventional GC.

			***Citrus limon*** **EO**					***Lavandula angustifolia*** **EO**					
			**Genuine**		**Spiked Samples**		**Com. Sample** **CB-1 EO**	**Genuine**		**Spiked Samples**		**Com. Sample** **CL-1 EO**	
	**I^t^sexp**	**I^t^slit**	**%**	**σ**	**%**	**σ**	**%**	**%**	**σ**	**%**	**σ**	**%**	**σ**
linalool	1100	1098	11.4	0.1	15.2	0.1	15.0	30.0	0.2	38.8	0.1	39.2	0.3
linalyl acetate	1260	1259	27.5	0.2	32.6	0.4	33.1	35.0	0.4	42.8	0.1	42.9	0.5
			***Melaleuca alternifolia*** **EO**										
			**Australian**		**Mixed Origins**		**Com. Sample** **CT-1 EO**						
	**I^t^sexp**	**I^t^slit**	**%**	**σ**	**%**	**σ**	**%**	**σ**					
4-terpineol	1178	1177	44.0	0.6	42.6	0.1	41.4	0.6					
α-terpineol	1190	1189	3.0	0.1	4.8	0.04	5.0	0.7					

**Table 4:** Absolute concentrations of the selected marker compounds in the investigated essential oils.

Essential Oil		(Linalool) (g/100 g)	σ	(Linalyl Acetate) (g/100 g)	σ	(4-Terpineol) (g/100 g)	σ	(α-Terpineol) (g/100 g)	σ
*C. limon*	genuine	10.5	0.2	25.9	0.4				
	5% spiked	9.6	0.1	23.8	0.6				
	20% spiked	7.1	0.3	21.8	0.2				
	50% spiked	5.0	0.1	14.4	0.3				
*L. angustifolia*	genuine	23.4	1.2	27.0	0.9				
	5% spiked	20.1	0.5	23.4	0.2				
	20% spiked	18.4	0.3	21.2	0.6				
	50% spiked	13.4	0.2	15.3	0.4				
	CL-2 EO	14.5	0.3	16.8	0.2				
*M. alternifolia*	genuine					44.2	2.7	8.5	1.5
	5% spiked					42.0	2.2	6.9	0.9
	20% spiked					33.5	1.8	3.5	0.2
	50% spiked					26.7	2.1	2.4	0.1

**Table 5:** Absolute concentrations of the enantiomers of linalool and linalyl acetate in both genuine and vegetable oil-spiked CL-2 EO.

Essential Oil		EC%	((*R*)-(−)-Linalool) (g/100 g)	σ	EC%	((*S*)-(+)-Linalool) (g/100 g)	σ
*L. angustifolia*	genuine	99.6	22.1	0.2	0.4	1.5	0.1
	5% spiked	99.6	19.2	0.2	0.4	1.3	0.1
	20% spiked	99.6	16.7	0.3	0.4	0.91	0.1
	50% spiked	99.6	12.3	0.2	0.4	0.62	0.08
	CL-2 EO	99.6	14.5	0.2	0.4	0.74	0.09
		**EC%**	**((*R*)-(−)-linalyl acetate)** **(g/100 g)**	**σ**	**EC%**	**((*S*)-(+)-linalyl acetate)** **(g/100 g)**	**σ**
	genuine	99.7	26.7	0.4	0.3	0.44	0.2
	5% spiked	99.7	23.1	0.2	0.3	0.34	0.2
	20% spiked	99.7	20.8	0.2	0.3	0.29	0.1
	50% spiked	99.7	15.1	0.1	0.3	0.21	0.06
	CL-2 EO	99.7	16.8	0.2	0.3	0.26	0.07

**Table 6:** List of the investigated EO and the selected marker compounds, together with their target ion (*m/z*), used for the quantitation.

Essential Oil	Botanical Name	Plant Part Used	Selected Marker Compounds	Target Ion
Bergamot	*Citrus limon* (L.) Osbeck	Peel	Linalool, linalyl acetate	71, 93
Lavender	*Lavandula angustifolia* Mill.	Aerial part	Linalool, linalyl acetate	71, 93
Tea tree	*Melaleuca alternifolia* (Maiden & Betche) Cheel	Leaves	4-terpineol, α-terpineol	71, 59

## Data Availability

Data is contained within the article or supplementary material.

## Supplementary Materials

The following are available online: Tables S1–S3. EC% of all enantiomeric compounds in the genuine essential oils investigated. Table S4. The equations for the calibration curves that were obtained with an external standard approach and used to quantitate the marker compounds, together with their correlation coefficients and the selected range of concentrations. Figure S1. The GC-MS extracted ions of linalool (*m/z* = 71) in a bergamot EO, with three consecutive extractions from a sample (on the left) and its linear decay diagram (on the right).

## Sample Availability

Not available.
