# Supplementary material for: Adulteration of Essential Oils: A Multitask Issue for Quality Control. Three Case Studies: Lavandula angustifolia Mill., Citrus limon (L.) Osbeck and Melaleuca alternifolia (Maiden & Betche) Cheel
Source: Molecules. 2021 Sep 16;26(18):5610. doi: 10.3390/molecules26185610 (PMC8471154; doi:10.3390/molecules26185610)
Supplement: Supplementary file 1 [file molecules-26-05610-s001.zip › molecules-1342948-supplyment material-original.pdf]

Table S1, Genuine Lavandula angustifolia EO enantiomeric composition

I^{t}sexp	I^{t}slit	**Compound**	**Percent Enantiomeric Composition**
1139	1133	(S)-(-)-camphor	9.0
1147	1141	(R)-(+)-camphor	91.0
1180	1174	(R)-(-)-linalool	93.8
1197	1189	(S)-(+)-linalool	6.2
1196	1192	(S)-(-)-borneol	17.8
1204	1200	(R)-(+)-borneol	82.2
1233	1231	(R)-(-)-linalyl acetate	99.4
1243	1237	(S)-(+)-linalyl acetate	0.6
1444	1454	(R)-(+)-germacrene D	9.3
1457	1462	(S)-(-)-germacrene D	90.7

Table S2, Genuine Citrus limon EO enantiomeric composition

I^{t}sexp	I^{t}slit	**Compound**	**Percent Enantiomeric Composition**
952	944	(R)-(+)-β-pinene	6.6
961	955	(S)-(-)-β-pinene	93.4
979	972	(R)-(+)-sabinene	15.5
993	988	(S)-(-)-sabinene	84.5
1060	1056	(S)-(-)-limonene	1.8
1071	1072	(R)-(+)-limonene	98.2
1181	1174	(R)-(-)-linalool	98.6
1198	1189	(S)-(+)-linalool	1.4
1233	1231	(R)-(-)-linalyl acetate	98.7
1243	1237	(S)-(+)-linalyl acetate	1.3
1399	1403	(1R,9S)-(-)-*trans*-β-caryophyllene	1E

Table S3, Melaleuca alternifolia EO enantiomeric composition

I^{t}sexp	I^{t}slit	**Compound**	**% enantiomeric composition (Australian)**	**% enantiomeric composition (Chinese)**
929	921	(R)-(+)-α-pinene	n.r.	n.r.
929	923	(S)-(-)-α-pinene	n.r.	n.r.
952	944	(R)-(+)-β-pinene	67.9	5.3
963	955	(S)-(-)-β-pinen	32.1	94.7
1020	1017	(R)-(-)-phellandrene	39.4	47.7
1024	1020	(S)-(+)-phellandrene	60.6	52.3
1060	1056	(S)-(-)-limonene	39.0	2.0
1077	1072	(R)-(+)-limonene	61.0	98.0
1250	1248	(S)-(+)-4-terpineol	67.2	42.2
1258	1253	(R)-(-)-4-terpineol	32.8	57.8
1302	1296	(S)-(-)-α-terpineol	24.2	23.0
1317	1309	(R)-(+)-α-terpineol	75.8	77.0

Table S4. Equations of the calibration curves used to quantitate the marker compounds together with their correlation coefficient and the selected range of concentration.

Compound	Calibration range (mg/mL)	Calibration curve equation	Correlation values
linalool	0.047 – 4.7	y = 65878209x + 12265089	0.9894
linalyl acetate	0.047 – 4.7	y = 71332322x + 13224402	0.9896
4-terpineol	0.5 - 5.0	y = 32642349x + 4197503	0.9989
α-terpineol	0.5 - 5.0	y = 20059684x + 2201700	0.9993
(R)-(-)-linalool	0.047 – 4.7	y = 94371962x + 17161963	0.9989
(S)-(+)-linalool	0.047 – 4.7	y = 94648975x + 17010288	0.9992
(R)-(-)-linalyl acetate	0.5 - 5.0	y = 90768850x + 13321659	0.9994
(S)-(+)-linalyl acetate	0.5 - 5.0	y = 90026414x + 7356410	0.9990
