# Supplementary figures and images for: Adulteration of Essential Oils: A Multitask Issue for Quality Control. Three Case Studies: Lavandula angustifolia Mill., Citrus limon (L.) Osbeck and Melaleuca alternifolia (Maiden & Betche) Cheel
Source: Molecules. 2021 Sep 16;26(18):5610. doi: 10.3390/molecules26185610 (PMC8471154; doi:10.3390/molecules26185610)

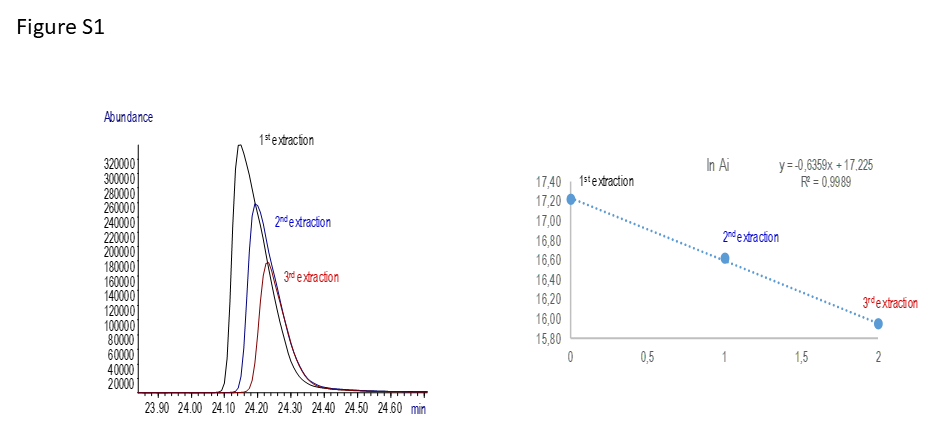

Supplement: Supplementary file 1 [file molecules-26-05610-s001.zip › molecules-1342948-supplementary-revised.tiff]
